# Mannosylated lipoarabinomannan in serum as a biomarker candidate for subclinical bovine tuberculosis

**DOI:** 10.1186/1756-0500-7-559

**Published:** 2014-08-21

**Authors:** Elise A Lamont, João Ribeiro-Lima, Wade Ray Waters, Tyler Thacker, Srinand Sreevatsan

**Affiliations:** Department of Veterinary Population Medicine, University of Minnesota, St. Paul, Minnesota USA; Department of Veterinary Biomedical Sciences, University of Minnesota, St. Paul, Minnesota USA; Faculdade de Medicina Veterinária, Universidade Lusósona de Humanidades e Tecnologias, Lisboa, Portugal; United States Department of Agriculture, National Animals Disease Center, Ames, Iowa USA; Veterinary Population Medicine and Veterinary Biomedical Sciences, College of Veterinary Medicine, University of Minnesota, 1971 Commonwealth Avenue, 55108 St. Paul, MN USA

**Keywords:** Bovine tuberculosis, Biomarker, *Mycobacterium bovis*, Cattle, Lipoarabinomannan, Diagnostics, Subclinical Infection, Serum

## Abstract

**Background:**

Early and unambiguous detection of bovine tuberculosis (bTB), a significant disease of cattle worldwide, is necessary to control the spread of infection to other animals and humans. Current testing strategies are laborious, time consuming and heavily reliant on host responses that do not distinguish bTB from other mycobacteria. We report the presence of a pathogen signature, liparabinomannan (LAM), as a potential biomarker for bTB infection.

**Findings:**

Fifty-five animals (uninfected [n = 33], bTb [n = 10] and exposed cases [n = 12]) from a well characterized bovine serum repository were screened for the presence of LAM using a commercially available ELISA. Analysis showed that LAM had a sensitivity of 100% and a specificity of 91.7% for bTB detection (bTB positive versus bTB exposed animals).

**Conclusion:**

LAM detection easily separated bTB infected animals from bTB exposed and negative controls. We propose that pathogen related markers, such as LAM, should be included with current testing strategies as a battery diagnostic for bTB.

**Electronic supplementary material:**

The online version of this article (doi:10.1186/1756-0500-7-559) contains supplementary material, which is available to authorized users.

## Findings

### Background

Successful control of bovine tuberculosis (bTB) is achieved through the interplay of best management practices and diagnostics [1]. Early detection of infection informs quarantine procedures to limit disease spread and guides changes to the management plan for the future of the herd. However, current bTB diagnostic tools are labor intensive, lack combined sensitivity and specificity, and rely heavily on host responses, which cross-react with other mycobacteria and may result in the unnecessary culling of uninfected animals [2–6]. Advances in *M. bovis* functional genome usage during infection identified the release of mycobacterial specific proteins, peptides, DNA and lipids that are shed or secreted into host fluids [7, 8]. We are now poised to capitalize on these biological processes to develop diagnostics that center on the presence of a pathogen signature. In a previous study, we utilized three *M. bovis* specific peptides (Pks5, MB1895c, and MB2515c) identified in bTB infected sera as biomarkers for detection of subclinical infection [9]. We showed that all three *M. bovis* peptides were capable of detecting bTB infected animals and Pks5 had little to no cross-reactivity to other mycobacterial species [9]. It is important to note that the next-generation of *M. bovis* diagnostics is likely to follow a battery testing model, which includes multiple pathogen signatures, such as peptides and lipids. Therefore, we hypothesized that the major mycobacterial cell wall glycolipid, mannosylated lipoarabinomannan (ManLAM), also circulates in the serum and may be utilized as a biomarker for bTB infection. We validated the presence of ManLAM in randomly selected samples from a well characterized cattle serum repository including confirmed cases of *M. bovis* (n = 10), bTB exposed (animals in contact with bTB positive cattle; n = 12) and negative controls (n = 33). Based on sensitivity and specificity analyses, we propose that ManLAM may aid current diagnostic tools for bTB infection.

## Methods

### Sample source

Field samples from bovine tuberculosis infected and exposed cases were generously provided by the National Veterinary Services Laboratory (NVSL; United States Department of Agriculture, Ames, IA) serum repositories. All field samples were collected from a single herd in California. Bovine tuberculosis disease status for each animal was validated using a combination of bacterial culture, antemortem caudal fold tests (CFT) and lesion histology at necropsy. Johne’s disease history for this herd was indeterminate. Bovine tuberculosis exposed cases were defined as animals that had contact with bovine tuberculosis infected animals but remained bovine tuberculosis negative (i.e. negative results for culture, CFT and histology). Negative controls were collected from a bovine tuberculosis free dairy herd in Minnesota. Negative controls tested negative for *M. bovis* (culture, histology, and CFT).

### Lipoarabinomannan Enzyme-linked immunosorbent assay (ELISA)

Randomly selected field sera samples (bTB positive n = 10 and bTB exposed n = 12), negative controls (n = 33), and negative controls spiked with either *M. tuberculosis* strain H37Rv purified mannosylated lipoarabionmannan (ManLAM) (BEI Resources; NR-14848) or *M. smegmatis* purified non-mannose-capped lipoarabinomannan (AraLAM) (BEI Resources; NR-14849) (Biodefense and Emerging Infections Research Resources Repository, NIAID, NIH) were diluted 1:5 in PBS and analyzed for the presence of LAM using the human LAM ELISA kit (Biotang, Waltham, MA) per manufacturer’s instructions. A LAM standard curve was included using kit controls and two-fold dilution series of ManLAM spiked sera. The standard curve was plotted using GraphPad Prism software (GraphPad Software, La Jolla, CA). The optical density was read at 450 nm with a wavelength correction at 570 nm. All samples were read in three wells. LAM ELISA was repeated twice.

### Statistical analysis

Optical densities for each animal were averaged across the replicates. Receiver operating characteristic (ROC) curves were compared for 1) positive versus within-herd negative exposed (exposed) and 2) positive versus negative controls using the area under the ROC curves (AUC). Optimal cutoff values were determined by maximizing specificity and sensitivity by plotting the true negative rate against the true positive rate. ROC curves and specificity-sensitivity plots were created in SPSS® (IBM Corp. Released 2013, IBM SPSS Statistics for Windows, Version 22.0, Amonk, NY).

## Results

**Figure 1:**
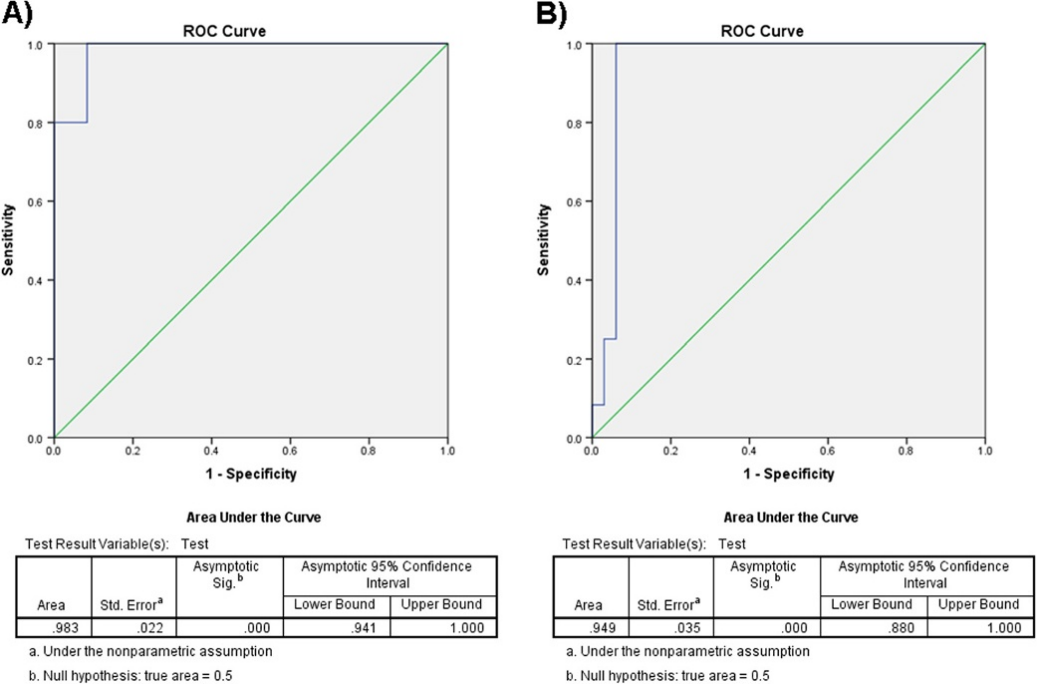
**Receiver operating characteristic (ROC) curves for LAM.** Each point on ROC curves is the fraction of **A)** bTB positive cattle (true-positive rate) versus the corresponding fraction of bTB exposed (false-positive rate) with an AUC of 0.983 and **B)** exposed bTB cattle (true-positive rate) versus negative controls (false-positive rate) with an AUC of 0.949.

**Figure 2:**
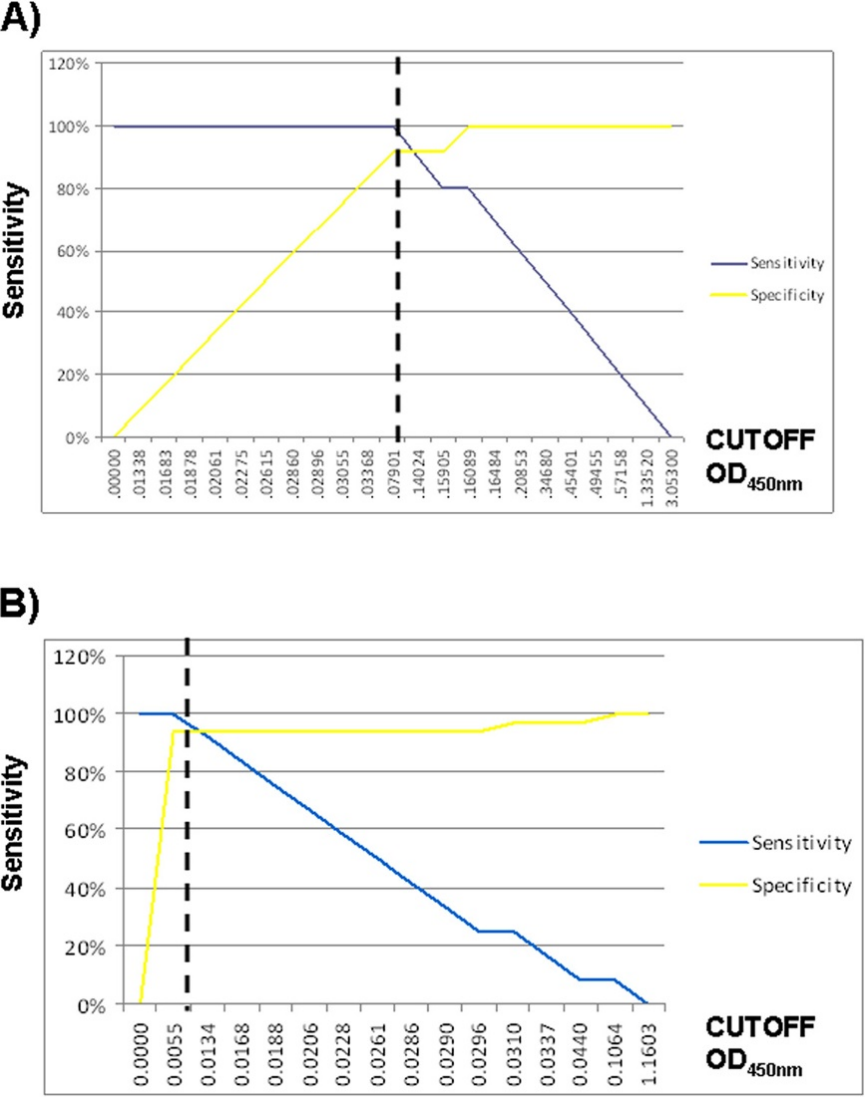
**LAM distinguishes bovine tuberculosis positive and negative controls and exposed animals.** The true negative rate and true positive rate using LAM as a biomarker were plotted against each other. The optimal cutoff value for **A)** bTB positive versus bTB exposed corresponded to O.D._450nm_ of 0.7901 (100% sensitivity and 91.7% specificity) and **B)** bTB exposed versus negative controls corresponded to O.D._450nm_ of 0.0055 (100% sensitivity and 93.9% specificity). Sensitivity = blue line and Specificity = yellow line.

During the establishment and progression of *M. bovis* infection, mycobacterial specific proteins and lipids, including those from the bacterial cell wall, are shed into host fluids. Several studies have shown the presence of lipoarabinomannan, a major cell wall glycolipid found on pathogenic mycobacteria, in sera and urine [10–13]. Therefore, we hypothesized that lipoarabionomannan may also be used as a biomarker to distinguish uninfected and *M. bovis* infected animals. Fifty-five animals (uninfected [n = 33], bTb [n = 10] and exposed cases [n = 12]) were screened for the presence of LAM using a commercially available ELISA. A LAM concentration curve was created and cross-reactivity to another glycolipid, non-capped lipoarabinomannan (AraLAM), found in environmental mycobacteria was assessed (Additional file 1). AraLAM was not discernable from the kit negative control (Additional file 1). Herds suspected of a bTB positive status will likely include contact animals (exposed) rather than pristine animals (i.e. uninfected and unexposed); therefore, ROC and AUC analyses compared bTB positive and negative controls against bTB exposed animals. The AUC showed a near perfect separation for bTB positive versus bTB exposed animals (0.983) and bTB exposed versus negative control animals (0.949) (Figure 1A and B, respectively). Five cutoff ODs (0.3368-0.16089 nm) that retained high sensitivity and specificity were identified in bTB positive and exposed comparison by ROC analysis. ROC analysis of bTB exposed versus negative controls determined 3 cutoff ODs (0.0055-0.068 nm). The true positive and negative rates were plotted against the OD cutoffs to determine optimal cutoff values. The optimal cutoff value was calculated at 0.7901 nm with a sensitivity of 100% and a specificity of 91.7% for bTB positive versus bTB exposed animals (Figure 2A). The optimal cutoff value to distinguish bTB exposed animals from negative controls was calculated at 0.0055 with a sensitivity of 100% and specificity of 93.9% (Figure 2B). Together these data suggest that the LAM ELISA can be effectively applied for rapid detection of *M. bovis* infection and exposure.

## Discussion

While detection systems utilizing host responses provided a first layer in identification of suspect diseased animals, they lack the needed specificity to eliminate cross-reactivity with other non-pathogenic mycobacteria or confusion with chronic illnesses of a non-infectious nature [3]. We have shown that mycobacterial peptides circulating in the serum accurately predict bTB infected from bTB negative and exposed animals [9]. We have extended our previous study to investigate another mycobacterial related component, LAM, likely present in serum of bTB infected animals, which may be used with additional pathogen signatures to provide a battery test for subclinical bTB. Traditionally, LAM is shed into the urine of infected hosts and is currently under investigation as a biomarker for human tuberculosis [10, 12, 13]. However, a recent study by Sakamuri et al. reported LAM coupled to high-density lipoprotein (HDL) in human tuberculosis positive sera [14]. It is important to note that LAM ELISA results may reflect exposure to environmental mycobacteria rather than show specificity for pathogenic mycobacteria. Therefore, we included sera spiked with AraLAM from *M. smegmatis* cultures and detected no significant changes to OD (Additional file 1). We show the presence of LAM correlates with bTb infection and is capable of separating infected from bTB exposed and negative animals. A single cutoff value applied to the true negative and positive rates showed LAM with a sensitivity of 100% and specificity of 91.7% for bTB positive versus bTB exposed animals (Figure 2). The identification of a glycolipid in infected serum also opens the possibility for identification of other pathogen related lipid biomarkers.

It is important to note that we analyzed a limited sample number for the presence of LAM. Although the results presented in this manuscript are promising for bTB diagnostics, a larger sample size will be required for validation. Furthermore, given the results reported by Sakamauri et al., pre-processing of sera involving HDL-nanodisc pulldown may be required to identify all bTB positive cases [14]. In addition to an increased sample size, sera from animals infected with other pathogenic mycobacteria, such as *M. avium* subsp. paratuberculosis, will need to be assessed to determine if cross-reactivity exists.

We propose that pathogen signatures, such as LAM, should be included in a two layer testing strategy for bTB. The first layer is a host related component in which the current strategies, newly identified host biomarkers, and LAM serve to identify suspected bTB positive animals such that a preliminary quarantine can be implemented [15]. The second layer of testing centers on a battery of circulating pathogen peptides to specifically distinguish bTB from other mycobacteria and chronic diseases [9].

## Conclusions

LAM detection easily separated bTB infected animals from bTB exposed and negative controls. Future studies should include a larger sera sample size to validate LAM as a biomarker for bTB. We propose that pathogen related markers, such as LAM, should be included with current testing strategies as a battery diagnostic for bTB.

## Availability of supporting data

The data sets supporting the results of this article are included within the manuscript and its additional files.

## Electronic supplementary material

Additional file 1: **Lipoarabinomannan concentration curve.** Only the positive control (Biotang kit-positive) and bovine sera spiked with LAM (0–300 ng/mL) showed a positive signal. Bovine sera spiked with non-capped lipoarabinomannan (AraLAM; *M. smegmatis)* did not react. All samples were conducted in triplicate. (PDF 59 KB)

## Abbreviations

bTB: Bovine tuberculosis
CFT: Caudal fold test
LAM: Lipoarabinomannan
ManLAM: Mannosylated lipoarabionmannan
AraLAM: Non-mannose-capped lipoarabinomannan
ELISA: Enzyme-linked immunosorbent assay
ROC: Receiver operating characteristic
AUC: Area under the ROC curve
ODs: Optical densities
HDL: High density lipoprotein.
