# Supplementary figures and images for: Mannosylated lipoarabinomannan in serum as a biomarker candidate for subclinical bovine tuberculosis
Source: BMC Res Notes. 2014 Aug 21;7:559. doi: 10.1186/1756-0500-7-559 (PMC4152573; doi:10.1186/1756-0500-7-559)

## Standard Curve-LAM ELISA

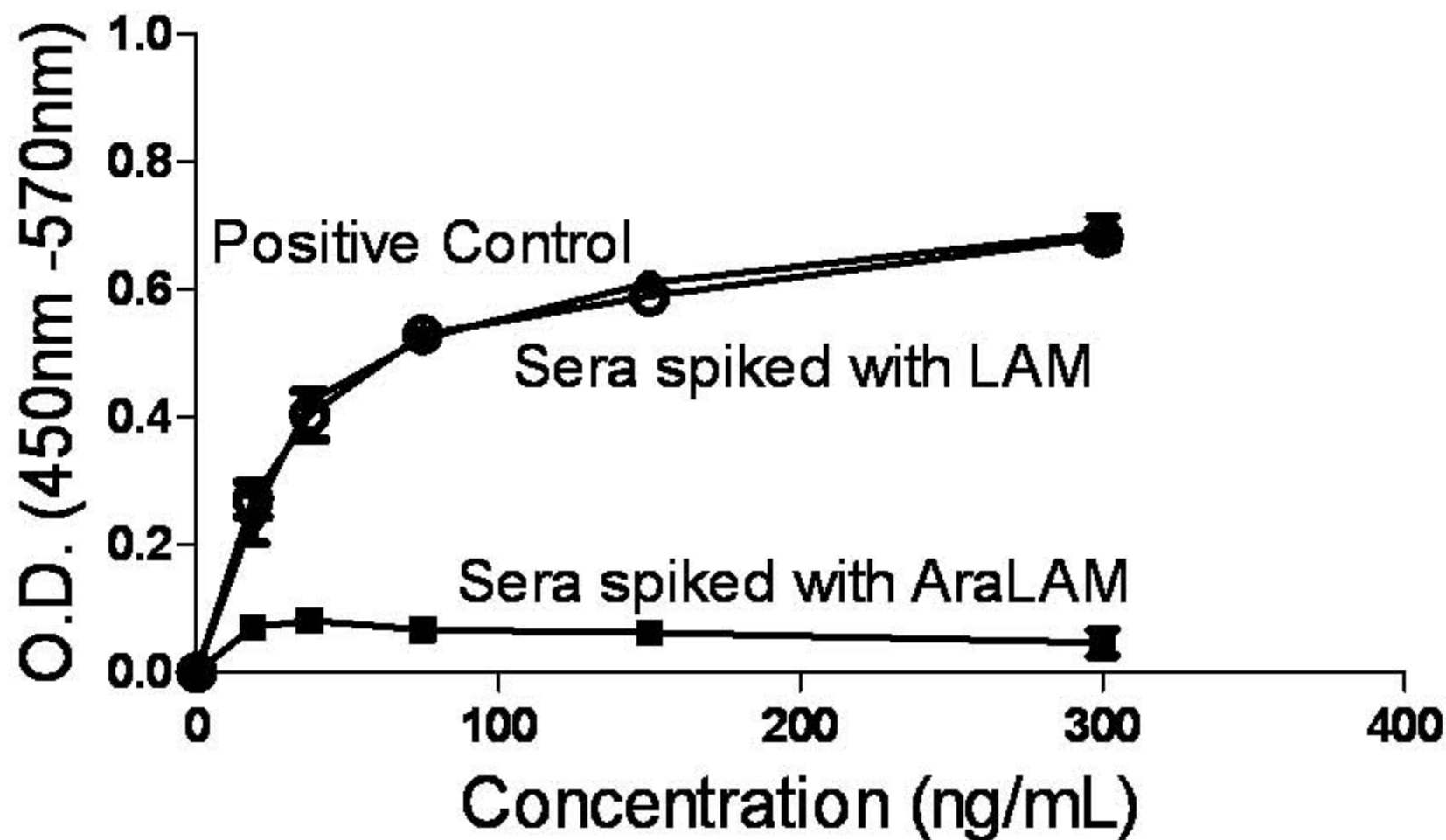

Supplement: Supplementary file 1 — Additional file 1: Lipoarabinomannan concentration curve. Only the positive control (Biotang kit-positive) and bovine sera spiked with LAM (0–300 ng/mL) showed a positive signal. Bovine sera spiked with non-capped lipoarabinomannan (AraLAM; M. smegmatis) did not react. All samples were conducted in triplicate. (PDF 59 KB) [file 13104_2014_3093_MOESM1_ESM.pdf]
